# Enhanced Recovery After Surgery (ERAS) Pathways for Aesthetic Breast Surgery: A Prospective Cohort Study on Patient-Reported Outcomes

**DOI:** 10.1007/s00266-023-03392-1

**Published:** 2023-06-01

**Authors:** Stéphane Stahl, Adelana Santos Stahl, You-Shan Feng, Arne Estler, Florian Buiculescu, Ana Cristina Seabra Robalo Gomes Jorge

**Affiliations:** 1 CenterPlast private practice, Bahnhofstraße 36, 66111 Saarbrücken, Germany; 2 Institute for Clinical Epidemiology and Applied Biometrics, Medical University of Tübingen, Tübingen, Germany; 3 Department of Diagnostic and Interventional Radiology, University Hospital of Tübingen, Tübingen, Germany; 4 Department of General, Visceral, Vascular, and Pediatric Surgery, Saarland University Hospital, Kirrberger Straße, 66421 Homburg/Saar, Saarland Germany

**Keywords:** Fast-track surgery, Fast recovery surgery, Enhanced recovery surgery, Breast augmentation, ERAS, Outpatient surgery

## Abstract

**Background:**

Patients’ expectations of an anticipated timeline of recovery and fear of anesthesia in aesthetic breast surgery have not been studied.

**Objective:**

This study aims to assess patient anxiety, expectations, and satisfaction after Enhanced Recovery after Surgery (ERAS) pathways for aesthetic breast surgery and the progress of postoperative recovery.

**Materials and methods:**

All consecutive patients who underwent aesthetic breast surgery between April 2021 and August 2022 were included in this single-center prospective cohort study. The ERAS protocol consists of more than 20 individual measures in the pre-, intra-, and postoperative period. Epidemiological data, expectations, and recovery were systematically assessed with standardized self-assessment questionnaires, including the International Pain Outcome Questionnaire (IPO), the BREAST-Q or BODY-Q, and data collection forms.

**Results:**

In total, 48 patients with a median of 30 years of age were included. Patients returned to most daily activities within 5 days. Eighty-eight percent of patients were able to accomplish daily activities sooner than expected. The time of return to normal daily activities was similar across all procedure types. There was no statistically significant difference regarding postoperative satisfaction between patients who recovered slower (12%) and patients who recovered as fast or faster (88%) than anticipated (*p*=0.180). Patients reporting fear of anesthesia in the form of conscious sedation significantly diminished from 17 to 4% postoperatively (*p*<0.001).

**Conclusion:**

Enhanced Recovery after Surgery (ERAS) pathways for aesthetic breast surgery are associated with rapid recovery and high patient satisfaction. This survey study provides valuable insight into patients’ concerns and perspectives that may be implemented in patient education and consultations to improve patient satisfaction following aesthetic treatments.

**Level of Evidence IV:**

This journal requires that authors assign a level of evidence to each article. For a full description of these Evidence-Based Medicine ratings, please refer to the Table of Contents or the online Instructions to Authors www.springer.com/00266.

**Supplementary Information:**

The online version contains supplementary material available at 10.1007/s00266-023-03392-1.

## Introduction

There is an ever-increasing pressure to perform surgeries in the ambulatory setting, primarily to improve cost-effectiveness. This trend has been reinforced by the COVID-19 pandemic [1]. Many studies have documented the safety and feasibility of ambulatory breast surgery in selected patients. Most of these studies have focused on outcomes, namely complications and readmissions [2, 3].

Understanding factors that enhance recovery and patient comfort, and increase patient satisfaction is a requisite for maintaining a successful practice. Enhanced Recovery after Surgery (ERAS), also known as fast-track surgery (FTS), consists of interdisciplinary multimodal perioperative interventions aimed to optimize the recovery process, including reducing hospital stay or avoiding hospitalization. The benefits of ERAS protocols have been studied in various surgical subspecialties [4]. Although research aiming at reducing opioid consumption and hospital stay dates to the 1990s, the term “enhanced recovery after surgery (ERAS)” has not gained much attention in cosmetic plastic surgery [5]. Little research has been done regarding functional recovery of patients after aesthetic breast surgery [6].

Patient satisfaction after surgery correlates with the fulfillment of patients’ presurgical expectations [7]. Unrealistic expectations predict an unsatisfactory outcome [8]. Patients believe that anesthesia and recovery are relatively simple and, therefore, restriction of activities after aesthetic surgery is not necessary [9, 10]. Divergence between actual experience and expectations impacts treatment adherence and the patients’ satisfaction [11]. Only one study examining patient satisfaction with postoperative recovery was identified in a systematic review of patient-reported outcomes following cosmetic surgery [12]. This gap of knowledge may lead physicians to undervalue the aspects of recovering.

This study’s objective is to investigate a) patients’ expectations about recovery timelines as well as concerns before undergoing aesthetic breast surgeries without general anesthesia and b) to evaluate recovery and satisfaction after enhanced ERAS pathways for aesthetic breast surgery.

## Material and Methods

### Patients

The local Ethics Committee gave their permission to this prospective study (project no. 144/21). Informed consent for prospective analysis was obtained. All consecutive patients who underwent aesthetic breast surgery from April 2021 to August 2022 were included in this study. Underage patients and patients requiring revision surgeries within the first week were excluded, as were patients with incomplete self-assessment questionnaires or incomplete clinical study reports. The ERAS protocol and anesthesia was performed as previously described by the authors [13]. All patients received 1000 mg acetaminophen and 1000 mg tranexamic acid PO 2 h before surgery, as well as dexamethasone 4 mg IV before starting the surgery (Table 1).

**Table 1:** Anesthetic protocol

Timing	Anesthetic protocol
Preoperative	At least 2 appointments for comprehensive preoperative counseling
	Perioperative instructions attached to the informed consent form and sent via e-mail before surgery
	Chlorhexidine showering on the day of surgery
	Abstain from solid food 6 h before surgery and clear liquids 2 h before surgery
2 h before surgery	Preemptive medication PO
	Acetaminophen 1000 mg
	Tranexamic acid 1000 mg
15 min before surgery	Dexamethasone 4 mg IV
	Propofol 5 mg/kg/h continuous IV infusion pump
	Alfentanil 0.5 mg IV before the intercostal block and when patient responds to pain stimuli
	Intercostal block
	20 ml Ropivacaine 0.375%, 4 mg dexamethasone, and epinephrine (1:100.000) per side
5 min before incision	Local anesthesia
	20–100 ml Lidocaine 0.1% with epinephrine (1:500.000) per side
Intraoperative	Stress-reducing strategies (communication skills, background music, professional and anticipating team)
	No monopolar electrocautery
	Warming blankets and warmed tumescent solutions
48 h after surgery	Preemptive analgesia PO
	Acetaminophen 500 mg and
	Metamizole 500 mg alternately every 3 h
	Pro re nata medication PO
	Tilidine/naloxone (50/4 mg) slow-release tablets
	Dimenhydrinate 150 mg
Postoperative	Ambulation within the first hour
	Discharge upon tolerance to food, voiding, ambulation without assistance, absence of intolerable pain, and absence of nausea
	Discharge letter including postoperative instructions, follow-up appointments
	Cellular telephone access to surgeon
	Routine follow-up call by the surgeon 12 h after the surgery
	Knee-high compression stockings for 10 days

The authors described preoperative counseling explicitly in previously published work [13]. Patients engaged in minimal work-related physical activity and were advised to take 7 days off work. Return to moderate aerobic physical activity or strenuous arm workout was discouraged before the fifth postoperative week. Comprehensive perioperative instructions were attached to the informed consent form and sent via e-mail before surgery.

### Surgery and Locoregional Anesthesia

All intercostal blocks were performed with ropivacaine 0.375%, 4 mg of dexamethasone, and epinephrine (1:100.000). Immediately after sedation, the surgeon injected 5 ml in the subcostal region for intercostal block between the third and the seventh ribs in a standard fashion [14]. For hemostasis and local anesthesia, lidocaine 0.1% with epinephrine (1:500.000) was injected into the incision sites (Table 1), observing previously described maximum dosages of 28 mg/kg [15]. Ten minutes after surgery, patients were allowed to drink and, in the absence of nausea, to eat afterward. Patients were given permission for discharge after surgery if they tolerated food, could urinate, walk alone, had no unbearable pain, and had no nausea. Patients were requested to have the assistance of a caregiver at home on the first night. Patients received a discharge letter including postoperative instructions, follow-up appointments, and the surgeons’ cell phone number. The surgeons made a postoperative follow-up phone call later the same day.

### Questionnaires and Clinical Study Reports

Clinical study reports were designed involving all members of the team as easy-to-use data collection forms with sufficient and unambiguous data. Satisfaction with the operative result was assessed with the BREAST-Q Augmentation, BREAST-Q Reduction/Mastopexy, or BODY-Q Chest module. Anxiety and satisfaction regarding anesthesia were assessed with 5 items from the validated International Pain Outcome Questionnaire (IPO) [16]. Items from the World Health Organization (WHO) International Classification of Functioning, Disability, and Health (ICF) were chosen based on expert opinions and literature research [17, 18]. The development of the questionnaire has been described elsewhere [13]. Primary and secondary outcome parameters and timing of assessments were:

- Structured self-assessment questionnaire for demographic data (gender, age, BMI, comorbidities, medication, and ASA score) completed before the first consultation.
- Patient-reported anxieties and satisfaction regarding anesthesia were assessed by the IPO administered before and the day after surgery.
- Patient-reported anticipated and actual recovery in the first postoperative week was assessed with a self-assessment questionnaire before and 10 days after surgery.
- Clinical study reports with descriptive data regarding surgery and anesthesia were assessed on the operation day by the circulating nurse.
- Patient-reported satisfaction with the operative result was assessed with the BREAST-Q Augmentation, BREAST-Q Reduction/Mastopexy or BODY-Q Chest module administered before the first consultation and at least 4 weeks postoperatively.

### Statistics

For continuous variables with a normal distribution, the mean and standard deviation (95% confidence interval) are presented, whereas for non-normal data, the median and interquartile range. The statistical analyses were carried out with SPSS Version 28 (SPSS Inc., Chicago, Illinois, USA). The correlations between the various parameters were evaluated using a Spearman correlation matrix. *P* ≤0.05 was used to define significance. We used the Wilcoxon rank-sum test to access group differences across nonparametric variables.

## Results

No patient dropped out, two were excluded due to operative revision, and one because of an incomplete questionnaire. Forty-eight patients with a median (IQR) of 30 (36–25) years of age were included in this study. Eighty-one percent of participants were women, and the median (IQR) BMI was 24 (26–22) kg/m^2^. Comorbidities, medication, and ASA score are presented in Table 2.

**Table 2:** Demographic data (age, BMI, comorbidities, medication, ASA score)

Age median (IQR)	30 (36–25)
BMI median (IQR)	24 (26–22) kg/m2
Sex	39 (81%) women
ASA I	34 (71%)
ASA II	14 (29%)
Comorbidities*	No comorbidities: 40 (83%)
	3 (6%) thyroid disease
	2 (4%) arterial hypertension
	2 (4%) asthma
	4 (8%) other
Medication*	No medications: 34 (71%)
	8 (17%) hormonal contraception
	3 (6%) levothyroxine
	2 (4%) antihypertensive drugs
	5 (10%) other
Profession (according to the International Standard Classification Of Occupations, ISCO-08):	5 (10%) skill level 1
	17 (35%) skill level 2
	9 (19%) skill level 3
	7 (15%) skill level 4
	7 (15%) not applicable
	3 (6%) not mentioned

*The total number does not sum to 48 (100%)

Nearly half of all surgeries performed were breast augmentations. Breast reductions comprised 35% and gynecomastia about 17% of all surgical interventions. Mastopexy was performed in 23% of patients. Sixty-nine percent of the surgeries involved additional procedures such as liposuction.

The median operating time was 02:12 (IQR: 02:49–01:36) hours. Gynecomastia corrections were the shortest procedures (median (IQR) 1:42 (1:52–1:09) hours) and breast reductions the longest (median (IQR) 2:37 (2:58–1:52) hours). The median (IQR) duration of breast augmentation (including implant and fat graft only, as well as hybrid breast augmentation) was 2:14 (2:49–1:35) hours.

The average doses of administered alfentanil and propofol were, respectively, 0.31 µg/kg/min (minimum 0.02, maximum 0.83 µg/kg/min) and 4.43 mg/kg/h (minimum 2.27, maximum 6.21 mg/kg/h).

### Satisfaction with Outcome

Our results show a statistically significant increase in satisfaction with breast appearance, as well as psychological and sexual well-being postoperatively (Table 3). There was an increase of 32 points (*p*≤0.001) in patient satisfaction with breasts, 18 points (*p*≤0.001) in psychosocial well-being, and 10 points (*p*≤0.001) in sexual well-being. Sixty-two percent of the respondents reported 100% satisfaction with the outcome.

**Table 3:** BREAST-Q scores and grouping of different surgical techniques are shown (Rasch transformed score range, 0–100). Higher scores indicate higher satisfaction

Surgery type	Total	Questionnaire	Satisfaction with breasts			Psychosocial well-being			Sexual well-being			Satisfaction with outcome
			Preoperative	Postoperative	*p* value**	Preoperative	Postoperative	*p *value**	Preoperative	Postoperative	*p *value**	
Breast augmentation	7	BREAST-Q	22 (33 –11) *	96 (100–82)*	*p*= <0.001	39 (58 –33)	89 (100–71)*	*p*= 0.003	40 (55 –20)	85 (100–68)*	*p*= 0.001	100 (100 –89)*
Augmentation mastopexy	1											
Hybrid breast augmentation	8											
Implant exchange and mastopexy	1											
Fat transfer	6											
Breast reduction and mastopexy	1	BREAST-Q Reduction/Mastopexy	44 (65–17)	94 (100 –88)*	*p*= 0.002	39 (63–28)	94 (100–75)*	*p*= <0.001	28 (39–15)	80 (100–69)	*p*= <0.001	100 (100–81)
Mastopexy and fat transfer	4											
Mastopexy	11											
Implant removal and mastopexy	1											
Gynecomastia	8		40 (95–15)	80 (89–13)	*p*= 0.046					80 (89–13)		
Total/Mean	48		22 (50–16)*	94 (100–81)*	*p*= <0.001	39 (59–31)	92 (100–75)*	*p*= <0.001	33 (46–20)	83 (100–66) *	*p*= <0.001	100 (100–84)*

All scores are median (IQR)

*Skewed variable

**Wilcoxon signed-rank test

### Apprehension Before and After Anesthesia

**Fig. 1:**
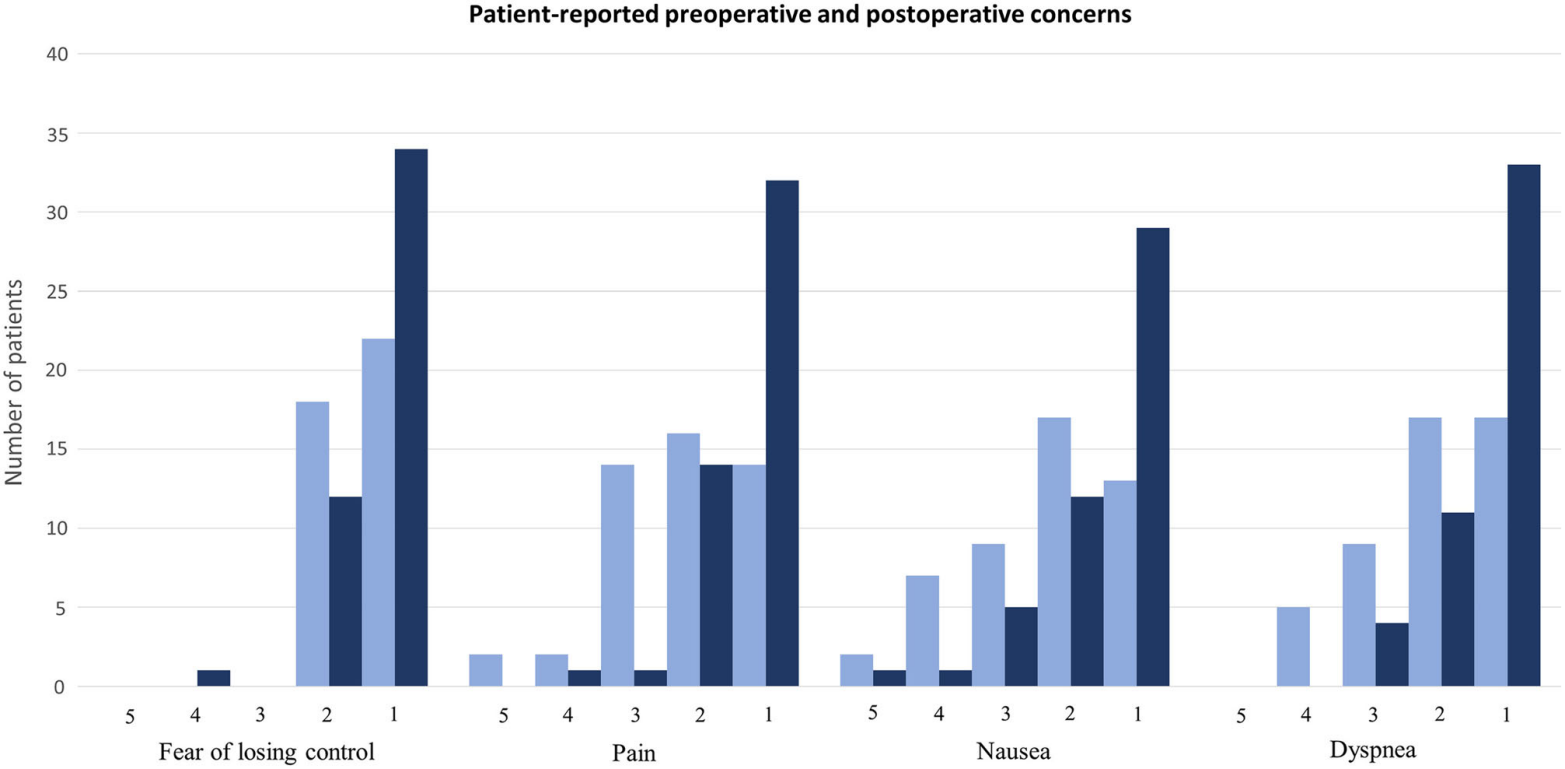
Patient-reported concerns about preoperative (light blue) as well as postoperative (dark blue) anxiety and fear of anesthesia (Before: “When I think about the upcoming anesthesia, I am concerned that...”; After: “If I needed this form of anesthesia in the future, I would be concerned that...”). Scale 1: not at all; 2: a little; 3: moderately; 4: strongly; and 5: very strongly

The greatest preoperative concerns of patients were reported using a 1 (not at all) to 5 (very strong) rating scale, and we observed a statistically significant reduction postoperatively (*p*≤0.001) in all categories (Fig. 1). In this series of outpatient aesthetic breast surgery under sedation, intercostal block, and tumescent anesthesia, most patients reported little or no concern about anesthesia (median (IQR) 2 (2–1)) and about feeling pain, nausea, or dyspnea (median (IQR) 2 (3–1)) before the procedure. This apprehension faded postoperatively, and patients revealed having no fear (median (IQR) was 1 (2–1) in all categories). Moderate, strong, or very strong concerns regarding the anesthesia in the form of conscious sedation were expressed by 17% of patients before the surgery and only by 4% of the patients after the surgery (Fig. 1).

There was no statistically significant correlation between preoperative anxiety and the dose of alfentanil or between anxiety and the dose of propofol required during surgery (*r*_*s*_= − 0.286) (Table 4). A power analysis for estimation of the sample size which compared the preoperative and postoperative concerns showed that the effect size of our study was extremely large (power 1.000, alpha 0.050).

**Table 4:** Spearman’s correlation of preoperative concerns and surgical and anesthetic parameters

	Satisfaction with breasts	Psychosocial well-being	Sexual well-being	BMI	Procedure duration (min)	Total alfentanil dose per patient (µg/kg/min)	Total propofol dose per patient (mg/kg/h)
Preoperative anxiety and fear of anesthesia:	rs= 0.119	rs= −0.179	rs= −0.123	rs= 0.152	rs= −0.082	rs= −0.090	rs= −0.286
	*p*= 0.435	*p*=0.283	*p*=0.462	*p*=0.303	*p*=0.578	*p*=0.609	*p*=0.095

### Anticipated and Actual Recovery

Patients returned to most daily activities within 5 days after surgery. Seventy-one percent of patients were able to perform most daily activities sooner than anticipated (*p*<0.001). Patients were able to wash themselves, climb stairs, and go for walks statistically significantly earlier than anticipated (Table 5). The first activities to be performed were walking around the house and climbing stairs. Patients were able to dress or undress and go for a walk outside on the following day, and to cook a meal and shower on the second postoperative day. Activities that required a longer recovery time to be accomplished were doing housework and shopping (on the fourth postoperative day) as well as driving a car (on the fifth day) (Table 5).

**Table 5:** Preoperative patients’ expectations regarding recovery and return to normal daily activities (“Please estimate your physical fitness in the first 7 days after surgery.”) and postoperative assessment of recovery and return to normal daily activities in the first 7 days after surgery (“When did you physically feel able to perform the following activities.”)

Days after surgery on which the patient anticipated (before surgery) and actually was able to perform the following activities (after surgery):															
Scale: 0: Day of Surgery; 1: 1st day; 2: 2nd day; 3: 3rd day; 4: 4th day; 5: 5th day; 6: 6th day; 7: 7th day; 8: After 1 week															
	To wash yourself (with shower plaster)			To dress or undress			To walk in the house/apartment			To climb stairs (1 floor)			To go for a walk outside (10 minutes)		
Surgery type	Preoperative	Postoperative	*p* value**	Preoperative	Postoperative	*p* value**	Preoperative	Postoperative	*p *value**	Preoperative	Postoperative	*p* value**	Preoperative	Postoperative	*P *value**
Breast Augmentation	2 (2 -1)	2 (3 -1)	*p*=0.239	1 (2–0)	2 (2–0) *	**p**=0.048	0 (1–0)*	1 (2–0)*	*p*=0.615	1 (2–0)	1 (1–0)*	*p*=0.302	2 (3–1)	2 (2–1)	*p*=0.161
Breast reduction/Mastopexy	1 (3–1)*	2 (4–1)	*p*=0.123	1 (2–0)*	1 (2–0)*	*p*=0.230	0 (1–0)*	0 (1–0)*	*p*=0.107	1 (2–0)*	0 (1–0)*	**p**=0.048	1 (3–1)*	1 (2–0)	*p*=0.088
Gynecomastia	1 (3–1)	3 (3–1)	*p*=0.063	1 (2–0)	1 (2–0)*	*p*=0.518	1 (2–0)*	0 (1–0)	*p*=0.705	1 (1–0)	0 (1–0)	*p*=1.000	1 (3–0)	1 (1–0)	*p*=1.000
Total/Median (IQR)	2 (3 - 1)*	2 (3 - 1)	**p**=0.014	1 (2 –0)*	1 (2 –0)*	*p*=0.323	0 (1–0)*	0 (1 - 0) *	*p*=0.438	1 (2 - 0) *	0 (1 - 0) *	**p**=0.042	2 (3 - 1) *	1 (2 - 1)	**p**=0.031

Days after surgery on which the patient anticipated (before surgery) and actually was able to perform the following activities (after surgery):												
Scale: 0: Day of Surgery; 1: 1st day; 2: 2nd day; 3: 3rd day; 4: 4th day; 5: 5th day; 6: 6th day; 7: 7th day; 8: After 1 week												
	To cook (a small meal for yourself)			To do housework (washing clothes, vacuuming the floor)			To go shopping (groceries)			To drive a car (up to 50 km)		
Surgery type	Preoperative	Postoperative	*P *value**	Preoperative	Postoperative	*P *value**	Preoperative	Postoperative	*P *value**	Preoperative	Postoperative	*P *value**
Breast Augmentation	2 (4 -1)	2 (3 -1)*	*p*=0.976	5 (7–3)	5 (5 - 4)	*p*=0.961	5 (7 - 3)	4 (5 - 4)	*p*=0.728	6 (8–3)	5 (8–2)	*p*=1.000
Breast reduction/Mastopexy	2 (5–1) *	2 (4–1) *	*p*=0.112	4 (8–2)	4 (5–2)	*p*=0.038	5 (8–3)	5 (6–2)	*p*=0.181	7 (8–2)	6 (8–3)	*p*=0.964
Gynecomastia	1 (2–1)*	1 (2–1)*	*p*=0.102	4 (7–2)	4 (5–3)	*p*=0.671	4 (6–2)	3 (4–3)*	*p*=0.317	2 (5–2)*	4 (8–3)	*p*=0.139
Total/Median (IQR)	2 (4–1) *	2 (3–1)e	*p*=0.659	4 (7–3)	4 (5–3)	*p*=0.110	5 (7–3)	4 (5–3)*	*p*=0.236	6 (8–2)	5 (8–3)	*p*=0.472

All scores are median (IQR)

*Skewed variable

**Wilcoxon signed-rank test

The speed of recovery did not correlate with the operating time (*r*_*s*_= 0.068, *p*=0.650) (Table 6). Neither the alfentanil nor the propofol dosages correlated significantly with the speed of recovery (Table 6). Recovery time was similar across categories, patients needed a median (IQR) of 2 (2–1), 2(3–1), and 2 (2–1) days after breast augmentation, reduction, and gynecomastia surgeries, respectively, to return to normal daily activities. Eighty-eight percent of patients required less time or exactly the same time to recover as anticipated preoperatively. However, patients with a slower recovery than anticipated were not less satisfied with the aesthetic result than patients whose recovery was as fast or faster than anticipated (rank-sum test: *p*=0.180). Power analysis showed a power of 0.983 to detect a difference of alpha 0.05 between preoperative concerns and postoperative recovery.

**Table 6:** Correlation of recovery and surgical and anesthetic parameters

	Satisfaction with breasts	Psychosocial well-being	Sexual well-being	Satisfaction with outcome	BMI	Procedure duration (min)	Total alfentanil dose per patient (µg/kg/min)	Total propofol dose per patient (mg/kg/h)
Recovery after surgery:	rs= − 0.057	rs= − 0.023	rs= − 0.017	rs= 0.025	rs= 0.015	rs= 0.068	rs= 0.158	rs= − 0.121
	*p*=0.708	*p*=0.890	*p*=0.912	*p*=0.935	*p*=0.920	*p*=0.650	*p*=0.372	*p*=0.496

## Discussion

We believe this is the first study to assess patients’ anxiety, expectations, satisfaction, and actual recovery time after ERAS pathways for outpatient aesthetic breast surgery.

Patients returned to most daily activities within 5 days (Table 5). A retrospective study of data collected between 1982 and 1990 suggested that reduction in surgical trauma enabled patients to lift objects up to 20 pounds, drive a car, return to work, and lie prone on their breasts 24 h after submuscular breast augmentation and general endotracheal anesthesia [19]. In a prospective study of patients after submuscular breast augmentations, patients resumed driving after 5.4 ± 4.1 (min: 1, max: 21) days after surgery. However, no details were given on the number and type of combined procedures and the type of anesthesia [6]. The literature pertaining to exercise after breast surgery shows no substantial evidence of adverse outcomes due to early postoperative exercise compared with delayed exercise [20]. Recent evidence suggests that exercise, including bench press, 1 week after breast augmentation does not increase complication rates [21]. Nevertheless, additional studies may be required to determine the optimum risk–benefit ratio between overly aggressive and overly cautious recovery.

Our findings corroborate that with optimal perioperative protocols complex surgical operations can be performed in an outpatient setting [22]. Studies suggesting that the operation time predicts the fitness of discharge do not consider the impact of general anesthesia on recovery [23], although a correlation between the type of anesthesia and the quality of recovery has been demonstrated [24, 25]. Under the standardized anesthesia protocol described herein, the range of operating times (min: 46 min; max: 293 min) did not correlate with the recovery time within the first postoperative week (Table 6). This may be explained by the overall low doses of propofol and alfentanil and their relatively short elimination half-life (558 ± 218 and 275 ± 94 minutes, respectively) [26]. Patients required a median (IQR) propofol dose of 4.97 (6.21–2.27) mg/kg/h, in contrast to the concentrations used in total i.v. anesthesia of 9 mg/kg/h as initial dose and 6 mg/kg/h as the maintenance dose [27]. As for alfentanil, the median (IQR) infusion rate was 0.33 (0.83–0.02) µg/kg/min, as opposed to the dose used in total i.v. anesthesia of 100 to 240 µg/kg/min [27]. The higher doses of propofol and alfentanil typically administered in general anesthesia difficult recovery, especially in the first 48 h, and therefore are more likely to require inpatient hospitalization postoperatively. The authors have previously shown that patients after aesthetic breast surgery according to the ERAS protocol described herein are recovering sooner than expected after general surgery (drinking, eating, and voiding within a median (IQR) of 0:45 (1:19–0:25) h, discharge within a median of 2:40 (3:43–1:58) h [13].

The risk of thromboses in cosmetic surgery, including breast and face surgeries, abdominoplasties, and liposuctions, is estimated at 0.9% and peeks at approximately 1 week after surgery [28]. Early mobilization and recovery are decisive to prevent thromboembolisms. Most patients in this study walked in their house or apartment and climbed stairs on the day of the surgery, which would not be possible in a hospital room, especially after general surgery. A significantly lower incidence of thromboembolism has been observed in a large published series of patients undergoing elective plastic surgery under total intravenous anesthesia [29]. Discussion on the association of thromboembolic events with operative time must acknowledge the potentially confounding effect of general anesthesia. Pain, nausea, and drowsiness are the most frequent causes for delaying discharge [30]. Evidence has been presented that opioid-sparing anesthesia improves recovery without compromising patient safety and pain control [13, 25].

Seventy-one percent of patients required as much time to recover as anticipated preoperatively or less. Evidence has been presented that preoperative information describing the recovery process reduces the length of hospital stay [31]. Well-informed patients present faster time to activity and recovery [38], are more satisfied with their overall outcome [41], and consume fewer opioids [32]. Communication gaps and unrealistic expectations increase the risk of postoperative complications [33]. Patients provided with adequate information and details about what to expect regarding the process of care and recovery are more satisfied with their overall outcome [34]. We, therefore, assume that providing the information described herein during preoperative counseling can further enhance recovery, increase satisfaction, and reduce opioid consumption. Because of the amount and importance of preoperative information, we recommend two appointments for comprehensive counseling and the use printed and digital formats.

Since stress and anxiety influence pain perception, immune function, and wound healing, the ERAS protocol included numerous measures to reduce anxiety. Structured follow-up calls, 12 to 24 h after ambulatory surgery potentially reduces patients’ self-reported pain and anxiety, and improves safety and outcomes by increasing compliance while reducing the traveling times, infrastructure needs, and contagion risk during the COVID-19 pandemic [35–38]. Having cellular telephone access has promising benefits for the patient–physician relationship [39] and is recommended to account for the risks of hematoma and the rare risk of lidocaine intoxication that may occur in the first 24 h after surgery [17]. Information and behavioral instructions have the potential to minimize stress and anxiety and improve postoperative outcomes [40]. Only six patients had a slower recovery than they had anticipated. Regarding the patients that recovered as fast or faster than they expected, the speed of recovery did not affect overall satisfaction (Table 6).

Preexisting mental health conditions, including depression, anxiety disorders, and substance abuse, have been noted to be important preoperative risk factors for adverse outcomes, resulting in increased likelihood of hospital-based acute care postoperatively [41]. Besides ASA III classification, which includes BMI ≥40, the strength of correlation and optimal cutoff values of patient selection parameters for outpatient care are unknown [42]. Since ambulatory surgery has many advantages, further studies are needed for appropriate patient selection (Table 7).

**Table 7:** Advantages of outpatient care

	Outpatient care versus overnight hospital stays
Ambulation	Outpatients walk around their house and climb stairs on the day of their surgery and can go for a 10min walk outside on the first postoperative day (Table 5)
Thrombosis	Outpatients have a smaller risk of thromboembolism [29]
Sleep	Outpatients have a better sleep [44]
Comfort	Outpatients benefit from a familiar environment and more privacy [44]
Opioid medication	Outpatients are less likely to become persistent opioid users [45]
Hospital-acquired bacterial infections	Outpatients have a smaller risk of drug-resistant infections [46]
Hospital-acquired COVID-19	Outpatients have a lower risk of nosocomial COVID-19 transmission [47]
Costs	Outpatient treatments are associated with lower costs [48, 49]
Risks/Surveillance	Routine monitoring of vital signs during overnight hospital stays is not effective and often inaccurate [50]
	A structured telephone/telemedicine follow-up call by surgeon 12 h postoperatively is advised [13]

This study must acknowledge some limitations. This study did not have matched controls of patients undergoing a standard perioperative model of care or standard general anesthesia. However, the empirical comparison of the ERAS pathway with the author’s prior experience shows that recovery is much shorter and less painful, especially in the first 24 h with the protocol described herein. Besides, because ERAS has been shown to be more effective when compared to standard protocol, doing so was deemed unethical. A response bias regarding recovery expectations can be ruled out since only questions regarding the earliest time to shower and drive after surgery were occasionally asked. We only present data from one institution; thus, the findings might not be generalizable to other institutions. Additionally, alternative anesthetic methods might not be compatible with the findings of this study. Because of the heterogeneity of professional activities, enhanced postoperative recovery is more reliably determined by the return to comparable activities of daily living. To satisfy the call for the use of reproducible and validated questionnaires, studies may be inclined to use established and validated instruments, compromising sensitivity and consistency [12]. We have used the BREAST-Q questionnaire to evaluate satisfaction after fat grafting to the breast, acknowledging issues regarding the consistency of several items (“The position of your implants on your chest (too high or too low)”; “How evenly your implants are positioned in relation to each other?”). Factors associated with hospital experience (room comfort, cleanliness, noise level, food service, bathroom comfort, etc.) are confounding factors affecting patient satisfaction with the outcome [43]. Since this study only involved ambulatory surgery, the impact of hospital experience on patient satisfaction can be ruled out. Additional liposuction performed in 69% of patients may account for an overestimation of residual pain, despite a high comfort due to intercostal blocks. However, patients with liposuction did not have significantly more pain than patients without liposuction (rank-sum test; *p* = 0.788). For optimal sensitivity and consistency, a questionnaire addressing important issues on recovery after aesthetic breast surgery has been developed for this research. The questionnaire is provided in the appendix for critical appraisal of validity or reproducibility. Recall bias can be excluded.

## Conclusion Fast Recovery Surgery

Patients and surgeons often expect aesthetic breast surgery to be performed under general anesthesia and have moderate apprehension about sedation, intercostal block, and tumescent anesthesia. The anesthetic techniques described herein are associated with quick recovery and high patient satisfaction. The results help improve patient education about their recovery. Patients, surgeons, and anesthesiologists should be familiar with the risks and advantages of various anesthetic techniques to make the best decision for the patient. The results encourage surgeons and anesthesiologists to closely collaborate to offer alternatives to general anesthesia in aesthetic breast surgery.

## Supplementary Information

Below is the link to the electronic supplementary material.

Supplementary file1 (DOCX 54 KB)
