# Supplementary material for: Enhanced Recovery After Surgery (ERAS) Pathways for Aesthetic Breast Surgery: A Prospective Cohort Study on Patient-Reported Outcomes
Source: Aesthetic Plast Surg. 2023 Jun 1;48(2):84–94. doi: 10.1007/s00266-023-03392-1 (PMC10234236; doi:10.1007/s00266-023-03392-1)
Supplement: Supplementary file 1 — Supplementary file1 (DOCX 54 KB) [file 266_2023_3392_MOESM1_ESM.docx]

Supplementary Appendix

Administered on the day of surgery

|  | When I think about the upcoming anaesthesia, I worry that... | not at all | a little | moderately | strongly | very strongly |  |
| --- | --- | --- | --- | --- | --- | --- | --- |
| 1.1 | ... I am at the **mercy of the anaesthesia** and have **no control** over myself. |  |  |  |  |  |  |
| 1.2 | ... I have **pain** during the operation |  |  |  |  |  |  |
| 1.3 | ... I will feel **nauseous** during the anaesthesia |  |  |  |  |  |  |
| 1.4 | ... I will have **shortness of breath** during anaesthesia |  |  |  |  |  |  |
| 1.5 | Would you be concerned about hospital-acquired infections if your surgery required you to stay in the hospital? |  |  |  |  |  |  |

|  | Physical exercise and activities of daily living are safe in moderation already after the first day of surgery.  Please estimate your physical fitness in the first 7 days after surgery.  Please mark the day after surgery on which you think that you will be physically able to perform the following activities. | | | | | | | | | | |  |
| --- | --- | --- | --- | --- | --- | --- | --- | --- | --- | --- | --- | --- |
|  |  | On the day of Surgery | 1^st^ day | 2^nd^ day | 3^rd^ day | 4^th^ day | 5^th^ day | 6^th^ day | 7^th^ day | After 1 week | Not applicable |  |
| 1.6 | To wash yourself (with shower plaster) |  |  |  |  |  |  |  |  |  |  |  |
| 1.7 | To dress or undress |  |  |  |  |  |  |  |  |  |  |  |
| 1.8 | To Walk in the house / apartment |  |  |  |  |  |  |  |  |  |  |  |
| 1.9 | To climb stairs (1 floor) |  |  |  |  |  |  |  |  |  |  |  |
| 1.10 | To go for a walk outside (10 minutes) |  |  |  |  |  |  |  |  |  |  |  |
| 1.11 | To cook (a small meal for yourself) |  |  |  |  |  |  |  |  |  |  |  |
| 1.12 | To do Housework (washing clothes, vacuuming the floor) |  |  |  |  |  |  |  |  |  |  |  |
| 1.13 | To go shopping (groceries) |  |  |  |  |  |  |  |  |  |  |  |
| 1.14 | To drive a car (up to 50 km) |  |  |  |  |  |  |  |  |  |  |  |

Administered on the day following the surgery

|  | If I needed this form of anaesthesia in the future, I would be concerned that... | not at all | a little | moderately | strongly | very strongly |  |
| --- | --- | --- | --- | --- | --- | --- | --- |
| 2.20 | ... I am at the **mercy of the anaesthesia** and have **no control** over myself. |  |  |  |  |  |  |
| 2.21 | ... I have **pain** during the operation |  |  |  |  |  |  |
| 2.22 | ... I will feel **nauseous** during the anaesthesia |  |  |  |  |  |  |
| 2.23 | ... I will have **shortness of breath** during anaesthesia |  |  |  |  |  |  |
|  |  |  |  |  |  |  |  |

Administered on the 10th days after surgery

|  | Physical exercise and activities of daily living are safe in moderation even after the first day of surgery.  We ask you to evaluate your **physical fitness** in the first 7 days after surgery.  Please mark the day after surgery on which you felt physically able to perform the following activities.  If you did not perform any of the activities, please indicate instead from which day you feel you could have performed the corresponding activity. | | | | | | | | | | |  |
| --- | --- | --- | --- | --- | --- | --- | --- | --- | --- | --- | --- | --- |
|  |  | On the day of Surgery | 1^st^ day | 2^nd^ day | 3^rd^ day | 4^th^ day | 5^th^ day | 6^th^ day | 7^th^ day | After 1 week | Not applicable |  |
| 3.1 | To wash yourself (with shower plaster) |  |  |  |  |  |  |  |  |  |  |  |
| 3.2 | To dress or undress |  |  |  |  |  |  |  |  |  |  |  |
| 3.3 | To Walk in the house / apartment |  |  |  |  |  |  |  |  |  |  |  |
| 3.4 | To climb stairs (1 floor) |  |  |  |  |  |  |  |  |  |  |  |
| 3.5 | To go for a walk outside (10 minutes) |  |  |  |  |  |  |  |  |  |  |  |
| 3.6 | To cook (a small meal for yourself) |  |  |  |  |  |  |  |  |  |  |  |
| 3.7 | To do Housework (washing clothes, vacuuming the floor) |  |  |  |  |  |  |  |  |  |  |  |
| 3.8 | To go shopping (groceries) |  |  |  |  |  |  |  |  |  |  |  |
| 3.9 | To drive a car (up to 50 km) |  |  |  |  |  |  |  |  |  |  |  |
